# 1513. Evaluating the Incremental Accuracy of HIV Self-Test Together with an App-Based Solution: A Secondary Data Analysis of Trial Data

**DOI:** 10.1093/ofid/ofad500.1348

**Published:** 2023-11-27

**Authors:** Ashlyn M Beecroft, Thomas Duchaine, Nora Engel, Chen Liang, Aliasgar Esmail, Keertan Dheda, Nitika Pai

**Affiliations:** McGill University, Montreal, Quebec, Canada; McGill University, Montreal, Quebec, Canada; Maastricht University, Maastricht, Limburg, Netherlands; McGill University, Montreal, Quebec, Canada; University of Cape Town Lung Institute, Cape Town, Western Cape, South Africa; University of Cape Town Lung Institute, Cape Town, Western Cape, South Africa; McGill University, Montreal, Quebec, Canada

## Abstract

**Background:**

According to the United Nations Programme on HIV/AIDS (UNAIDS), in 2019, two out of every seven new human immunodeficiency virus (HIV) infections, globally, were among young people aged 15 to 24 years old. HIV self-testing (HIVST) is a convenient strategy that helps increase knowledge of HIV serostatus in the young. In 2012, the Food and Drug Administration (FDA) approved the OraQuick In-Home HIV Test with a reported sensitivity of 92%. Digital supports such as applications (Apps) and websites, in conjunction with oral self-tests, have demonstrated a high feasibility, acceptability, and impact, yet data on accuracy with digital supports remain largely unexplored.

**Methods:**

We performed a secondary data analysis of a quasi-randomized trial of oral HIVST with HIVSmart! conducted in South African township populations (2017-2019). We hypothesized that HIVSmart! guided interpretation increased test accuracy. We evaluated the diagnostic accuracy of the HIVSmart! guided interpretation of an oral self-test result against the reference standard (dual Elisa and HIV RNA). Stored picture of a self-test result was uploaded by the participants.

**Results:**

Accuracy data from (n=1489) HIVST participants versus reference standard (2 rapid tests and HIV RNA) demonstrated:

Sensitivity: 95.52% (95% CI: 94.48%-96.56%)

Specificity: 99.93% (95% CI: 99.79%-100.00%)

Positive Predictive Value: 99.22% (95% CI: 98.78%-99.67%)

Negative Predictive Value: 99.57% (95% CI: 99.24%-99.90%)

**Conclusion:**

With the App, we noticed an improved sensitivity to 95.5% (from 92% with self-tests without the use of the App), specificity high at 99%, together with high positive and negative predictive values. Findings demonstrate that Smart-App based digital interpretation removed subjectivity and increased accuracy of test result interpretation, together with recording and storage of data for monitoring purposes. Findings suggest that Smart-App based readers could be useful adjuncts to improve the accuracy estimations of self-tests, translating to increased trust and confidence in self-tests.

**Disclosures:**

**All Authors**: No reported disclosures

